# A novel marker relationship between carotid intima–media thickness and disease activity score-28 in patients with rheumatoid arthritis: human endothelial cell-specific molecule-1

**DOI:** 10.3906/sag-1806-60

**Published:** 2019-12-16

**Authors:** Göksel TUZCU, Ali Uğur USLU, Ayça TUZCU, Rabia Aydoğan BAYKARA, Ahmet OMMA, Adem KÜÇÜK

**Affiliations:** 1 Department of Radiology, Aydın Atatürk State Hospital, Aydın Turkey; 2 Department of Internal Medicine, Yunus Emre State Hospital, Eskişehir Turkey; 3 Department of Biochemistry, Faculty of Medicine, Aydın Adnan Menderes University, Aydın Turkey; 4 Department of Physical Medicine and Rehabilitation, Malatya Education and Research Hospital, Malatya Turkey; 5 Division of Rheumatology, Department of Internal Medicine, Numune Education and Research Hospital, Ankara Turkey; 6 Department of Rheumatology, Meram Faculty of Medicine, Necmettin Erbakan University, Konya Turkey

**Keywords:** Human endothelial cell-specific molecule-1, rheumatoid arthritis, atherosclerosis, carotid intima–media thickness, marker

## Abstract

**Background/aim:**

Human endothelial cell-specific molecule-1 (endocan) is a marker of vascular endothelial dysfunction that may be used in the evaluation of inflammatory-associated atherosclerotic lesions. Endocan may be a marker for the evaluation of atherosclerosis and disease activity in rheumatoid arthritis (RA) patients.

**Materials and methods:**

We included 39 RA patients assessed according to the American College of Rheumatology/European League Against Rheumatology 2010 diagnostic criteria and recruited 30 age- and sex-matching healthy subjects for the control group.

**Results:**

Endocan values were 14.11 ± 3.27 for the RA patients and 12.10 ± 2.92 for the controls. The endocan values of the patients were significantly higher than those of the control group (P = 0.009). In the correlation analysis, endocan showed a significantly positive correlation with disease activity score-28 (r = 0.386, P = 0.029) and carotid intima–media thickness (cIMT) (r = 0.419, P = 0.008). Linear regression analysis revealed that there was an independent relationship between endocan and cIMT (P = 0.029).

**Conclusion:**

Endocan can be a marker for early atherosclerosis and disease activity in RA patients.

## 1. Introduction 

Rheumatoid arthritis (RA) is a systemic inflammatory, autoimmune disease with a prevalence of 0.5%–1% in the general population. Its etiology is unknown. Symmetrical joint involvement and erosion and deformities in joints due to synovial inflammation are seen during the clinical course of the disease [1,2]. Although the pathogenesis of this disease is not clear, it is thought that genetic and environmental factors play a critical role. It is more commonly seen in females than in males [3]. In RA patients atherosclerosis (ATHS) and ATHS-associated cardiovascular diseases are among the principal causes of mortality and morbidity [4].

Responses originating from inflammatory cells, mediators, and vascular cells in the pathogenesis of ATHS are of great importance. Human endothelial cell-specific molecule-1 (endocan) is secreted by vascular endothelial cells, especially by the inflammatory endothelial cells [5,6]. Endocan, which is detectable in serum, has recently become an essential marker of angiogenesis and endothelial cell activation [6–8]. In the assessment of subclinical ATHS and the progression in ATHS-associated cardiovascular diseases carotid intima–media thickness (cIMT) is an easy, noninvasive indicator [9]. Studies investigating the relationship between endocan levels and cIMT indicate that endocan can be used to assess cardiovascular diseases in rheumatologic diseases such as Behçet’s disease [10] and systemic lupus erythematosus [11].

Our aim in the present study was to investigate serum endocan levels in RA patients and research the clinical usability of the disease activity and atherosclerotic process evaluation.

## 2. Materials and methods

Our study was conducted in Malatya State Hospital rheumatology and physical therapy and rehabilitation clinics prospectively covering the period between June 2016 and March 2017. We included 39 RA patients assessed according to the American College of Rheumatology/European League Against Rheumatology (ACR/EULAR) 2010 diagnostic criteria and recruited 30 age- and sex-matched healthy subjects for the control group [12]. The primary clinical and laboratory characteristics and the disease activity score-28 (DAS-28) of the patient group and the basic laboratory characteristics of the control group were recorded. The disease activity score of the cases was calculated with the DAS-28 index stated below [13]. DAS-28 = (0.56 × √number of tender joints) + (0.28 × √number of swollen joints) + (0.7 × erythrocyte sedimentation rate (ESR)) + (0.014 × visual pain scale).

Patients with systemic diseases such as diabetes mellitus, hypertension, coronary artery disease, chronic obstructive pulmonary disease, and cancer and those with a smoking and alcohol use history were not included. The study was approved by the Ethics Committee of Ankara Numune Education and Research Hospital, Turkey. Written and verbally informed consents of all participants were obtained.

### 2.1. Carotid intima–media thickness measurement

Imaging was conducted using a high-resolution ultrasound machine (Logiq S6; General Electric, Milwaukee, WI, USA) with a 12-MHz mechanical sector transducer in all cases. The examination was performed while the patient was in the supine position and his or her neck was turned away from the side to be examined. Intima–media thickness (IMT) measurements were obtained from plaque-free areas and distant from both main carotid arteries (ACA) and 1 cm before the bifurcation. The measurements were performed by the same researcher with the same device. Three measurements were recorded from both ACAs and the average values were calculated [9]. 

### 2.2. Human endothelial cell-specific molecule-1 (endocan) measurement

Coagulated blood samples were collected from both the patient and the control group after 12-h fasting. After appropriate centrifugation, the samples were stored at –80 °C until testing. The clinical data and blood samples were collected over a 10-month period. The concentration of endocan was assessed by the enzyme-linked immunosorbent assay (ELISA). Serum endocan levels were measured using ELISA kits (Uscn Life Science Inc, Wuhan, PR China). The ELISA process was performed according to the manufacturer’s instructions. Absorbance was evaluated at 450 nm via the ELISA reader [11].

### 2.3. Statistical analysis

All statistical analyses were performed with the Statistical Package for the Social Sciences (SPSS) 24.0 (SPSS Inc., Chicago, IL, USA). Descriptive statistics were presented as arithmetic mean ± standard deviation, and categorical variables were stated with numbers and percentages (%). Results that did not follow normal distribution are expressed as median (range) values. Student’s t-test assessed the significance of the mean differences between groups. Abnormally distributed data were analyzed using the Mann–Whitney U test. Differences were assessed by chi-square test for categorical variables. Relationships between variables were tested using Pearson’s correlation analysis. Linear regression analysis was performed to determine the independent relationship between cIMT and endocan levels and other parameters. P values less than 0.05 were regarded as significant.

## 3. Results 

**Figure 1:**
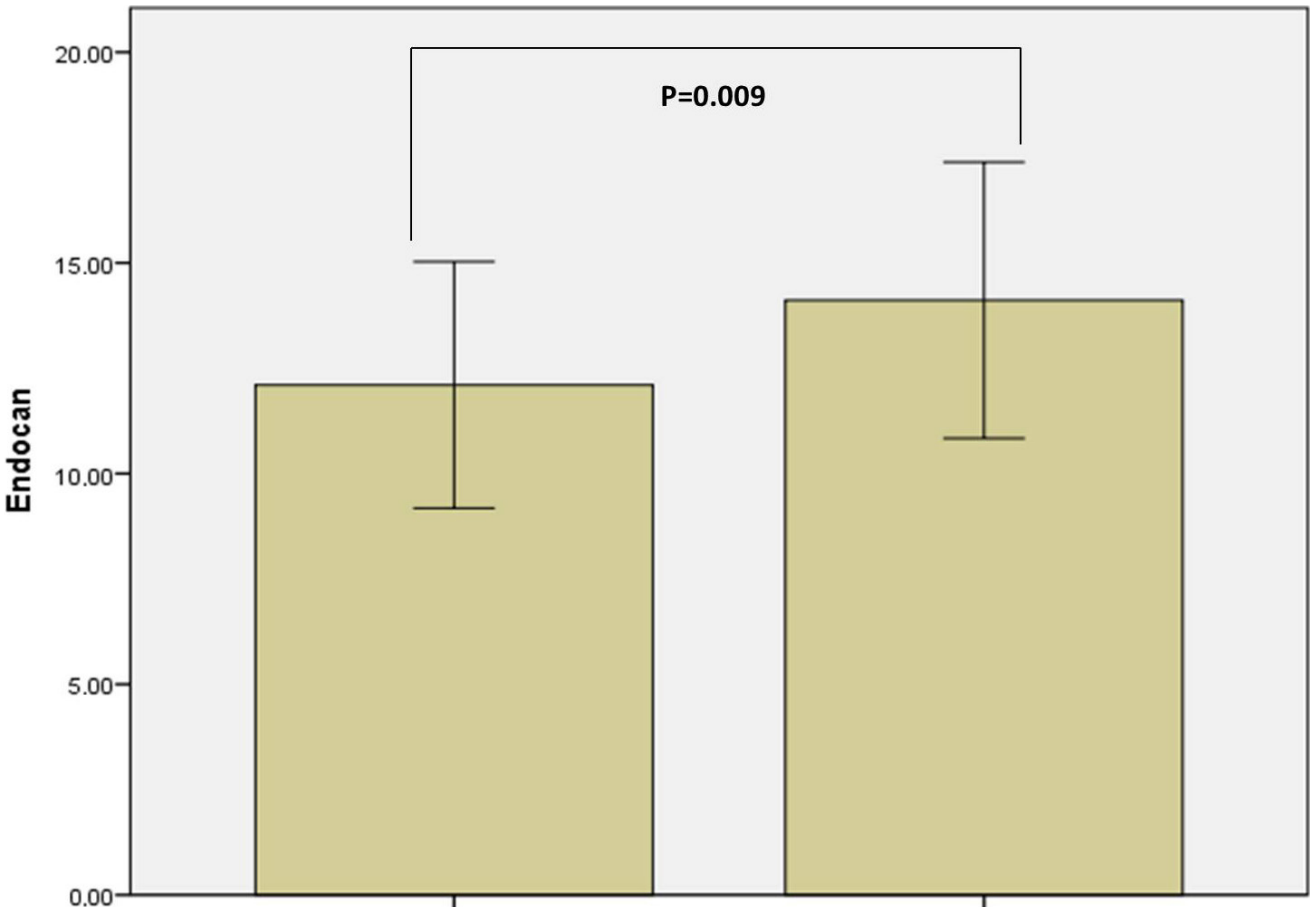
Endocan levels.

There was no statistically significant difference in terms of age, sex, or body mass index (BMI) values between the RA patients and the controls (Table 1). Endocan values were 14.11 ± 3.27 for the RA patients and 12.10 ± 2.92 for the controls. The endocan values of the patient group were significantly higher than those of the control group (P = 0.009) (Table 1; Figure 1). While ESR and total cholesterol (TC) values were significantly higher in the RA patients (P = 0.006, P = 0.001, respectively), high density-lipoprotein cholesterol (HDL-c) values were lower (P < 0.001). There were no significant differences between the two groups in terms of C-reactive protein, triglyceride (TG), or low density-lipoprotein cholesterol (LDL-c) (P = 0.123, P = 0.804, P = 0.637, respectively). The main clinical and laboratory characteristics of the patients and the controls are shown in Table 1. 

**Table 1:** The sociodemographic and laboratory parameters in the two groups.

	RA patients (n = 39)	Controls (n = 30)	P value
Age (years)	47.7 ± 8.8	43.0 ± 10.8	0.057
Sex (M/F), n (%)	33 (84.6)/6 (15.4)	21 (70)/9 (30)	0.238
BMI (kg/m^2^)	28.0 ± 4.3	27.1 ± 5.1	0.443
Hb (g/dL)	13.44 ± 1.44	13.94 ± 1.78	0.220
WBC (10^3^/L)	6.9 ± 2.9	7.1 ± 1.7	0.698
ESR (mm/h)	27.00 (5.00–75.00)	15.00 (3.00–56.00)	0.006
CRP (mg/dL)	0.29 (0.10–2.04)	0.11 (0.10–2.54)	0.123
TC (mg/dL)	191.00 (121.00–389.00)	165.00 (118.00–249.00)	0.001
TG (mg/dL)	120.00 (57.00–408.00)	125.00 (44.00–293.00)	0.804
LDL-c (mg/dL)	109.50 (66.10–280.00)	111.80 (66.30–156.90)	0.637
HDL-c (mg/dL)	42.12 ± 8.18	50.43 ± 10.18	<0.001
cIMT (mm)	0.63 ± 0.17	0.51 ± 0.12	0.001
Endocan	14.11 ± 3.27	12.10 ± 2.92	0.009

RA, rheumatoid arthritis; BMI, body mass index; cIMT, carotid intima–media thickness; TC, total cholesterol; TG, triglyceride; LDL-c, low density-lipoprotein cholesterol; HDL-c, high-density lipoprotein cholesterol; ESR, erythrocyte sedimentation rate; CRP, C-reactive protein; Hb, hemoglobin; WBC, white blood cell counts.

**Figure 2:**
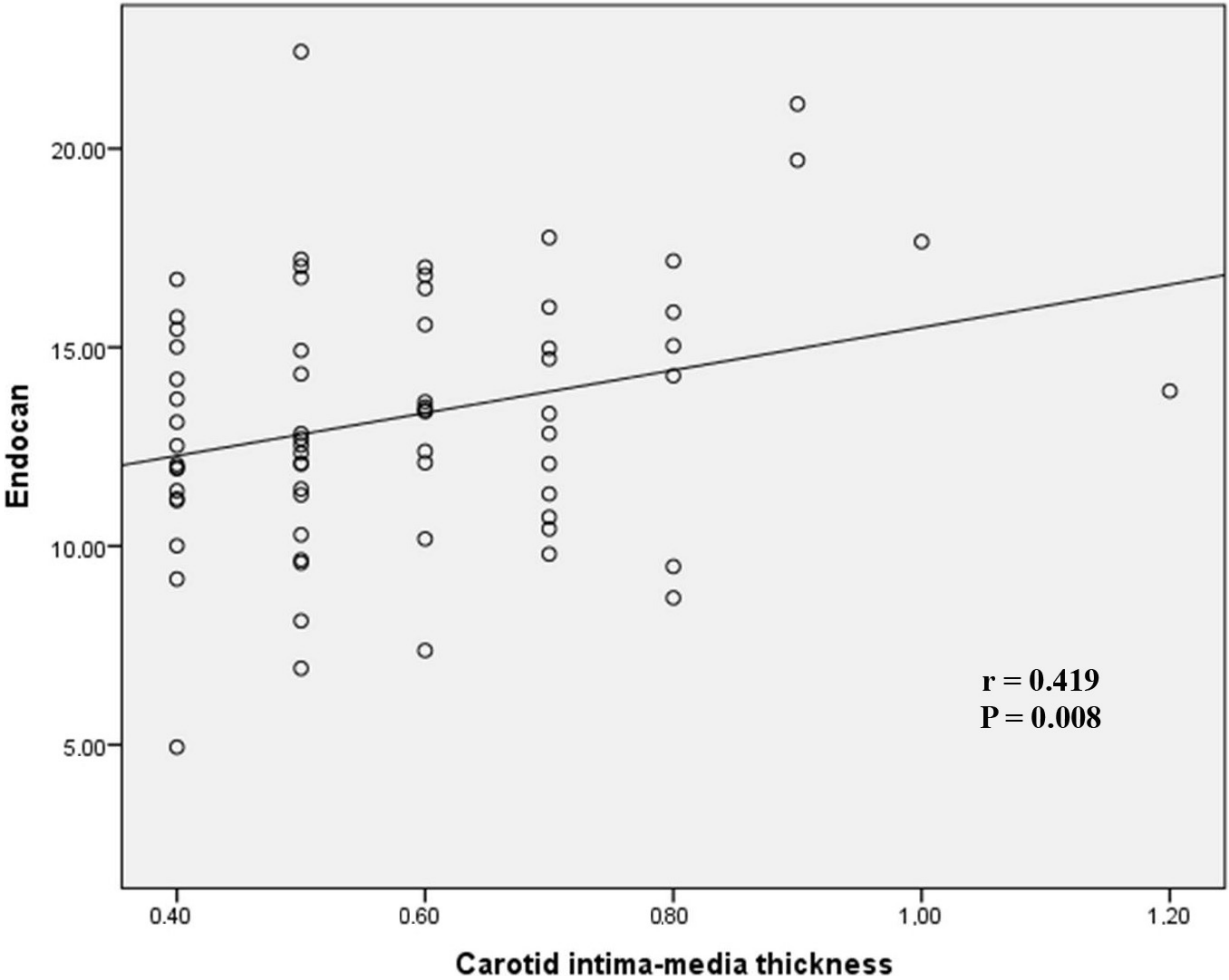
Endocan levels correlation with carotid intima–media thickness.

**Figure 3:**
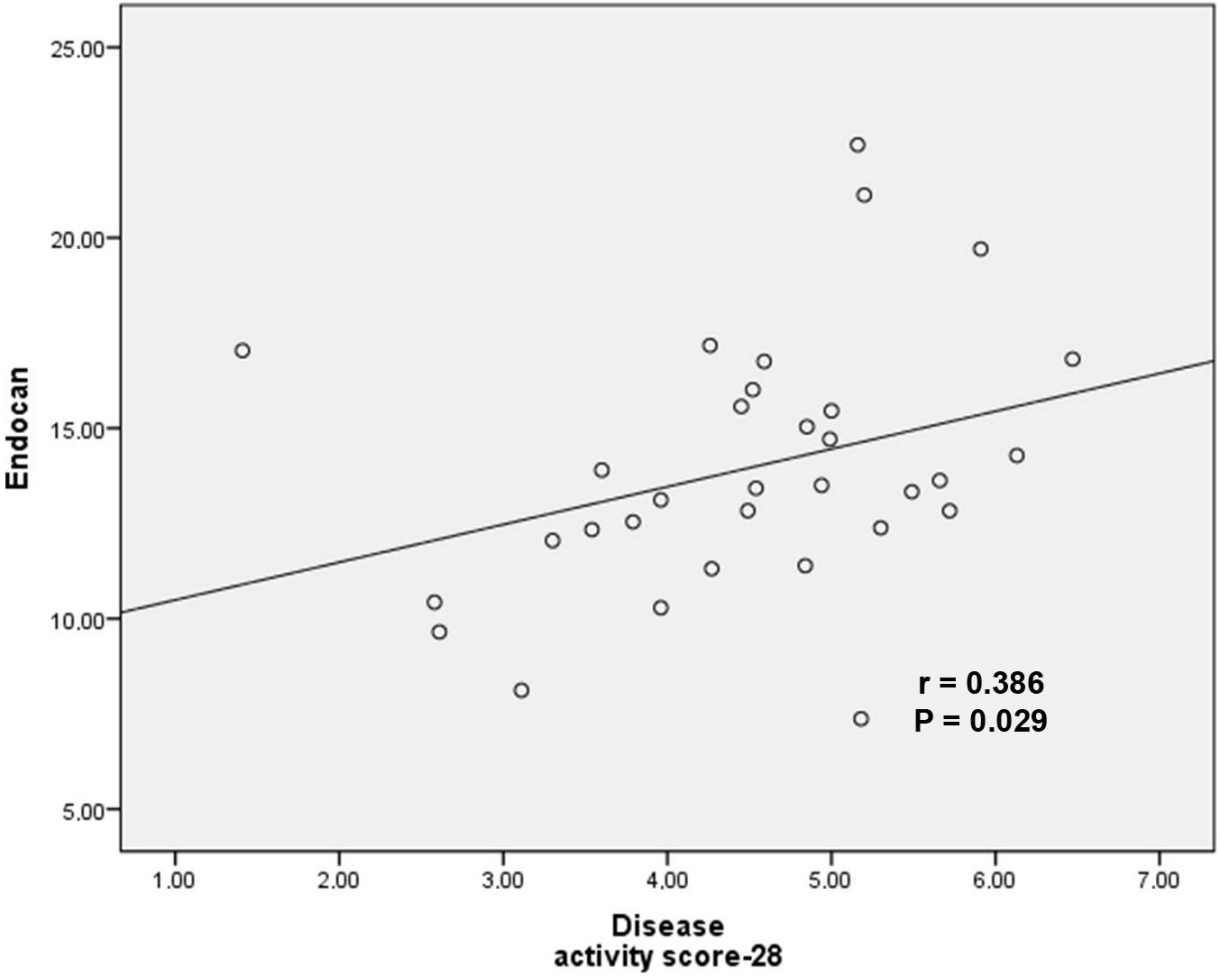
Endocan levels correlation with disease activity score-28.

In the correlation analysis, endocan showed a significantly positive correlation with age (r = 0.344, P = 0.032), DAS-28 (r = 0.386, P = 0.029), and cIMT (r = 0.419, P = 0.008) (Figures 2 and 3). There was no correlation with BMI (r = 0.249, P = 0.126), TC (r = 0.094, P = 0.567), HDL-c (r = 0.077, P = 0.942), TG (r = 0.115, P = 0.484), or LDL-c (r = 0.161, P = 0.329). There was no relation between the serum endocan levels and the age at diagnosis (r = –0.101, P = 0.569) or duration of disease (r = 0.041, P = 0.818) in the RA patients. All of the results of the correlation analysis are shown in Table 2. Furthermore, no correlation was seen between endocan levels and rheumatoid factor (r = –0.063, P = 0.751) or anticyclic citrullinated peptide (r = –0.140, P = 0.397) in the RA patients. The linear regression analysis revealed that there was an independent relationship between endocan and cIMT (P = 0.029). The linear regression analysis is presented in Table 3. There was no difference between endocan levels and treatment modalities (steroid, tumor necrosis factor inhibitors, and disease-modifying antirheumatic drugs). 

**Table 2:** Endocan and correlation analysis of other parameters in the RA patients.

	Endocan	
	r value	P value
Age	0.344	0.032
BMI	0.249	0.126
Hb	–0.059	0.722
WBC	0.008	0.968
ESR	0.115	0.487
CRP	–0.083	0.616
TC	0.094	0.567
HDL-c	0.077	0.942
TG	0.115	0.484
LDL-c	0.161	0.329
DAS-28	0.386	0.029
cIMT	0.419	0.008
Disease duration	–0.101	0.569
Symptom duration	0.041	0.818

RA, rheumatoid arthritis; BMI, body mass index; ESR, erythrocyte sedimentation rate; CRP, C-reactive protein; TC, total cholesterol; HDL-c, high-density lipoprotein cholesterol; TG, triglycerides; LDL-c, low-density lipoprotein cholesterol; Hb, hemoglobin; cIMT, carotid intima–media thickness; WBC, white blood cell counts; DAS-28, disease activity score-28.

**Table 3:** Linear regression analysis for cIMT in the patients with RA.

Independent variables	Beta regression coefficient	P value
Age	0.473	0.003
BMI	0.080	0.571
FPG	–0.087	0.555
Creatinine	0.404	0.026
LDL-c	–0.088	0.508
HDL-c	0.133	0.331
Endocan	0.133	0.029

RA, rheumatoid arthritis; cIMT, carotid intima–media thickness; BMI, body mass index; FPG, fasting plasma glucose; HDL-c, high-density lipoprotein cholesterol; LDL-c, low-density lipoprotein cholesterol.

## 4. Discussion

In the present study, the relationships between serum endocan levels and cIMT and DAS-28 levels were evaluated in patients with RA. Our findings showed that serum endocan and cIMT levels were higher in the patients with RA compared to the controls. Serum endocan levels of the RA patients had a positive correlation with cIMT and DAS-28, which is used in the assessment of disease activity.

In RA patients, cardiac involvement can be seen in the form of endocarditis, myocarditis, pericarditis, arrhythmia, and valvular involvement. Besides these, cardiovascular events related to ATHS and ATHS are important causes of mortality and morbidity [14–16]. Although the process of the development of ATHS is not fully understood in RA patients, the factors involved in its etiology and the interactions between them are quite complex [15]. These complex interactions include the risk caused by the systemic diseases in the first place (such as diabetes mellitus, hypertension, and metabolic syndrome), and cigarette smoking and physical inactivity are also included. In addition, either positive or negative medical treatments (such as disease-regulating drugs, steroids, and tumor necrosis factor inhibitors) have an important impact on the disease activity and the ATHS process [15,16]. Together with these, the changes in the glucose and lipid metabolism and insulin resistance, which are the consequences of both medical treatments and the inflammatory process, have significant effects [15–17]. This whole complex process results in an increased risk of cardiovascular diseases and ATHS development in RA patients.

In the evaluation of ATHS, cIMT is an easy, cheap, and reliable indicator. The progression of subclinical ATHS and cardiovascular diseases in RA patients can be assessed with cIMT and increasing cIMT is known to be associated with cardiovascular diseases [9,18]. The study by Uslu et al. [18] showed that cIMT was higher in RA patients when compared to their controls. Tutoğlu et al. [19] have shown that cIMT was higher in RA patients than in the controls in their study. In another study, Vazquez-Del Mercado et al. [20] reported that cIMT was higher in RA patients compared to their controls and was correlated with DAS-28, which is used to assess disease activity.

ATHS development begins with endothelial dysfunction; the process continues with the increase in chemokine production, increase in expression of adhesion molecules, and increased endothelial permeability [8,21]. Monocytes and low-density lipoproteins (LDLs) pass into the subendothelial tissue. LDLs are oxidized there and phagocytized by macrophages to form foam cells. In the meantime, proinflammatory cytokines are involved by causing thickening of the vessel wall and plaque formation [16,22]. Endocan has been proposed as a vascular endothelial dysfunction biomarker and has been assessed as a marker that can be used in the evaluation of ATHS lesions [8,23]. 

Endocan is a 50-kDa soluble proteoglycan secreted by human vascular endothelial cells. The proinflammatory cytokines Interleukin-1, tumor necrosis factor alpha, and microbial lipopolysaccharides and vascular endothelial growth factor play an important role in the secretion of endocan [23]. There are studies performed on the association between endocan levels and rheumatologic diseases. Balta et al. [10] showed that endocan levels in Behçet’s patients were higher than those in their controls and endocan levels could be used to evaluate disease activity. In the study by Icli et al. [11] serum endocan levels in systemic lupus erythematosus patients were higher than those in the controls. In the same study, they stated that there was also an association with cIMT. In another study, by Ozalper et al. [24], serum endocan levels in familial Mediterranean fever patients were higher than those in the controls.

Our study is the first to evaluate the relationship between endocan levels and cIMT and DAS-28 in RA patients. The presence of systemic inflammation and oxidative stress in RA patients plays an active role in the development of ATHS [21,23]. This process suggests that there may be cIMT elevation and changes in endocan levels in RA patients. Moreover, in our study the higher endocan and cIMT values in the RA patients compared to the controls and the positive correlation between serum endocan levels and cIMT and DAS-28 suggest that endocan may be a marker for the evaluation of disease activity and ATHS in RA patients. 

With the existing data, our study has some limitations. These include the low number of patients in the study population and the single center experience, as well as the lack of assessment of the differences between groups that could possibly be generated based on the DAS-28 value. 

On the other hand, for evaluating endocan as a marker for early ATHS, it is necessary to conduct a study comparing RA patients with and without ATHS or RA patients with and without clinically important increased cIMT.

In conclusion, this study suggests that endocan may be a marker for early ATHS and disease activity in RA patients. We think there is a need for multicenter prospective studies involving a higher number of patients. 
